# Sensory specificity and speciation: a potential neuronal pathway for host fruit odour discrimination in *Rhagoletis pomonella*

**DOI:** 10.1098/rspb.2016.2101

**Published:** 2016-12-28

**Authors:** Cheyenne Tait, Srishti Batra, Sree Subha Ramaswamy, Jeffrey L. Feder, Shannon B. Olsson

**Affiliations:** 1 Department of Biological Sciences, University of Notre Dame, South Bend, IN 46556, USA; 2 Naturalist-Inspired Chemical Ecology, National Centre for Biological Sciences, Tata Institute of Fundamental Research, GKVK Campus, Bellary Road, Bangalore 560065, India

**Keywords:** ecological speciation, host shifts, electrophysiology, phytophagous insects, chemosensory system, host choice

## Abstract

Behavioural changes in habitat or mate choice can trigger population divergence, leading to speciation. However, little is known about the neurological bases for such changes. *Rhagoletis pomonella* (Diptera: Tephritidae) is a model for ecological speciation via host plant shifts. Within the past 180 years, *Rhagoletis* flies infesting hawthorn (*Crataegus* spp.) shifted to attack domesticated apple (*Malus pumila*). The two populations differ in their olfactory preferences for apple versus hawthorn fruit. Here, we looked for patterns of sensory organization that may have contributed to this shift by characterizing the morphology, specificity and distribution of olfactory sensory neurons (OSNs) on the antennae of *Rhagoletis* responding to host fruit and non-host volatiles. Of 28 OSN classes identified, two colocalized OSN pairs were found that specifically responded to the major behavioural attractant and antagonist volatiles for each fly population. A reversal in the response of these OSNs to fruit volatiles, either through a switch in receptor expression between these paired neurons or changes in neuronal projections in the brain, could therefore account for the behavioural difference between apple and hawthorn flies. The finding supports the hypothesis that relatively minor changes in olfactory sensory pathways may contribute to rapid host shifting and divergence in *Rhagoletis*.

## 1. Introduction

Ernst Mayr once said that behaviour is ‘the pacemaker of evolution’ [1]. By this he meant that changes in behaviour affecting an organism's preference for different habitats or mates may act as catalysts for population divergence, potentially leading to speciation. Changes in habitat choice may be particularly important for organisms that are ecological specialists mating within preferred environments [2]. In this case, differences in habitat choice directly translate into differences in mate choice, generating reproductive isolation between populations. The reduction in gene flow can facilitate the evolution of habitat-related performance and survivorship differences and further the speciation process.

Habitat choice may play an important role in triggering adaptive divergence for phytophagous insects, the most diverse group of organisms on the planet [3]. Most phytophagous insects are specialized on one or a limited number of host plants, which represent temporal and spatial resource islands [4]. It is therefore critical for phytophagous insects to rapidly distinguish their preferred hosts among a diversity of alternative plants less suitable for feeding, mating and oviposition. One important way that insects accomplish this is through differences in their olfactory preferences for chemical cues associated with their host plants [5,6].

Despite the significance of habitat choice for adaptive evolution and speciation, relatively little is known about its neurophysiological basis. The apple maggot fly, *Rhagoletis pomonella*, is a model for rapid ecological divergence in phytophagous insects via host plant shifts. These flies originally infested the fruit of downy hawthorn (*Crataegus mollis*) in the eastern USA, but in the last 180 years shifted to attack introduced, domesticated apple (*Malus pumila*) [2,7]. *Rhagoletis* flies mate exclusively on or near the fruit of their host plants [7,8]. Differences in host plant choice therefore result in assortative mating and prezygotic isolation between populations of apple and hawthorn flies in nature [9].

Previous studies have confirmed that apple and hawthorn flies differ in allele frequency for many genes across the genome [10–12], consistent with their status as partially reproductively isolated ‘host races’, the hypothesized first stage of ecological speciation with gene flow. Laboratory and field studies have also established that the host races differ in their behavioural responses to volatile compounds emitted from the surface of ripening apple versus downy hawthorn fruit [13–16]. Moreover, it has been shown that these olfactory cues are used by flies to locate and discriminate between apple versus downy hawthorn trees for mating and oviposition. Thus, olfactory discrimination for different fruit volatiles generates reproductive isolation between apple and hawthorn flies.

The behavioural difference between apple and hawthorn flies could involve neurological changes at one or more levels of organization in the chemosensory system. At the peripheral level, insects detect volatiles using an ensemble of compound-specific olfactory sensory neurons (OSNs) on their antennae [17,18]. In flies, there are generally two to four different types of OSNs housed together (colocalized) in stereotyped combinations within hair-like cuticular structures on the antenna called sensilla [19,20]. Each receptor protein expressed by a particular OSN binds to a specific subset of odorants in the environment. The binding triggers the neurons to fire, sending information to a specific region of the brain known as the antennal lobe, which then projects to higher brain regions, ultimately leading to a behavioural response [21].

Genetic and behavioural analyses of F_2_ and backcross hybrids between apple and hawthorn *Rhagoletis* flies have suggested that their olfactory divergence is based on differences at only a few genetic loci [22], implying a small number of changes in the neural network having large effects on behaviour. OSNs are the first point where such changes in the chemosensory network could occur, with changes in the number, selectivity, sensitivity or targeting of OSNs to the central nervous system shaping downstream processing that ultimately evokes host choice behaviour. Such has been seen in *Drosophila sechellia*, and similarly in *Drosophila erecta*, where a single type of olfactory receptor neuron responding to key host volatiles increased in number after a host shift [23,24]. By contrast, in the European corn borer, *Ostrinia nubilalis*, the specificities of two colocalized OSNs reversed their response to two isomer components (E and Z) of the sex pheromone blend, as the species diverged into two strains responding to opposite pheromone blend concentrations [25–29].

The key fruit volatiles inducing behavioural differences between apple and hawthorn flies have been identified and synthetic blends developed that result in the same level of response in flight tunnel assays as whole fruit extracts (table 1) [13–16]. Apple and downy hawthorn fruit blends almost completely differ in the behaviourally active volatiles that flies respond to (table 1). Moreover, the major volatile attractant of apple flies in the apple blend, butyl hexanoate, acts as an antagonist to hawthorn flies at apple-like concentrations [14]. By contrast, the major volatile attractant of hawthorn flies in the downy hawthorn blend, 3-methyl-1-butanol, acts as a behavioural antagonist to apple flies [14,33]. A change in neuronal sensitivity or specificity to these key volatiles could thus not only be involved in the evolution of preference for apple, but also in the avoidance of downy hawthorn fruit seen in the apple race.

**Table 1.** Synthetic host fruit volatile blends. Shown are the proportions of behaviourally active compounds comprising apple (*Malus pumila*) and different hawthorn species blends, including those for downy hawthorn (*Crataegus mollis*), blueberry hawthorn (*C. brachyacantha*), green hawthorn (*C. viridis*), eastern mayhaw (*C. aestivalis*), western mayhaw (*C. opaca*), hybrid mayhaw (*C. rufula*) and southern red hawthorn (*C. mollis* var. *texana*). Data were compiled from [13,15,16,30–32].

volatile	downy haw	apple	green haw	blueberry haw	eastern mayhaw	western mayhaw	hybrid mayhaw	southern red haw
butyl butyrate		10	19.5	9	12	6	5	45
propyl hexanoate		4	1.5		1	6	1	0.3
butyl hexanoate	0.01	37	24	16.8	25	26	23	20
hexyl butyrate		44	24	16.8	9	12	6	14
pentyl hexanoate		5	2.5		2		3	0.6
3-methyl-1-butanol	4		5	0.6	2	44	1	0.4
butyl acetate				50	47		57	9
pentyl acetate				3.5	2	6		
isoamyl acetate	1.5						3	
isoamyl butyrate								1.5
isoamyl hexanoate								0.5
ethyl acetate	94.3							
dmnt	0.07		20.5					
di-hydro-beta-ionone	0.1							0.2
1-octen-3-ol			0.5	0.3				
hexyl propionate							1	
butyl propionate								5.5

Previous studies of olfactory divergence in *R. pomonella* have suggested that the apple and hawthorn host races do not differ in their number or classes of antennal sensilla [34]. However, these studies did not resolve details of individual OSN specificity, sensitivity and organization within sensilla. Thus, a targeted study at the level of individual OSNs is necessary to determine whether changes in specific OSNs could impact behavioural preference and speciation. For example, a few neurons responding to key apple and downy hawthorn volatiles could serve as sensory pathways upon which a small change in neural response may have a large impact on behaviour. To investigate such possibilities, we first morphologically and physiologically characterize the different classes of OSNs present in *Rhagoletis* using scanning electron microscopy and single sensillum recording (SSR) to a panel of 76 different odorants. We then compare the overlap in neuron ensembles responding to the different apple and hawthorn host fruits, and their patterns of organization both within sensilla and across the antennae. Finally, we discuss how our findings may allow for relatively simple changes in neural pathways to facilitate behavioural divergence and speciation in *R. pomonella*.

## 2. Material and methods

### (a) Insects

Apple race flies were obtained as pupae from a laboratory line maintained at the New York State Agricultural Experiment Station in Geneva, NY, USA and at the USDA-ARS Appalachian Fruit Research Station, Kerneysville, WV, USA. Adult hawthorn race flies were reared from collections of larval-infested fruit sampled at a field site in Grant, MI, USA using standard *Rhagoletis* husbandry methods [35]. Flies were maintained on a 15 L : 9 D light cycle at 25°C and 65% humidity on the artificial diet of sugar and yeast [36]. Individuals were used for scanning electron microscopy at approximately 14 days post-eclosion and for SSRs 2–30 days post-eclosion.

### (b) Scanning electron microscopy

Scanning electron microscopy (SEM) was used to examine the antennae of three male and three female apple race flies to determine the type of sensilla present on the periphery of *R. pomonella*. Whole heads with intact antennae were prepared and mounted as described elsewhere [37]. Images were obtained using a LEO 1450 VP scanning electron microscope (Zeiss, Wetzlar, Germany).

### (c) Electrophysiology

#### (i) Single sensillum recordings

Insects were prepared for SSR following *Drosophila* protocols described in detail elsewhere [38–40]. For initial identification of OSN response classes, SSR was performed on a total of 50 female apple fly adults. The recording electrode was inserted into the base of the target sensilla of a fly to form a stable electrical contact and high signal-to-noise amplitude spikes were visualized and recorded using Autospike (Ockenfels Syntech, Kirchzarte, DE, USA).

Responses were recorded to a panel of 76 different host and non-host compounds in random order (see below and electronic supplementary material, table S1 for details concerning the panel). The neuronal action potentials obtained from sensilla were quantified using Autospike and distinguished as separate neurons based on top-to-top spike amplitudes. Responses were determined from the difference in number of spikes 1 s before and after the stimulus. A response of less than 15 spikes s^−1^ (approx. 10% of the highest response in the study at 145 spikes s^−1^) was considered ‘no response’, as in previous studies [40,41]. If no chemical provoked a response from any neuron in a sensillum, that sensillum was removed from analysis. SSR was later performed with a reduced set of 20 chemicals on six female hawthorn race flies using the same methods above to verify whether similar neuron classes exist in both races, as asserted previously [34].

The sensitivities to varying concentrations of the key compounds butyl hexanoate and 3-methyl-1-butanol were examined for the OSNs in two sensilla types newly identified in this study and designated b7 and b9 (see below). Concentration-response studies were conducted on 10 apple and 10 hawthorn race flies, presenting odour stimuli in increasing concentrations from 10^−8^ to 10^−2^ w v^−1^. Recordings were controlled for solvent effects by subtracting out the control response to hexane. We normalized responses across the concentrations within each neuron by dividing by the highest response in the sequence. Normalized responses were averaged and graphed as a scatter plot, with a logistic line of best fit added (regression, curve estimation, logistic, version 21, IBM SPSS). We compared host race normalized responses using *t*-tests at each dosage within each neuron type.

#### (ii) Chemicals and chemical delivery

A panel of 76 different chemicals were used as stimuli to identify neuronal classes (electronic supplementary material, table S1), including odorants from blends attractive to the *R. pomonella* host races (table 1) [13,30–32], and diagnostic odorants used to characterize OSNs in *Drosophila* [17] and *Bactrocera invadens* [42]. The full set was reduced to 20 diagnostic chemicals for the validation study with the hawthorn race.

All chemicals were serially diluted from pure substances to 10^−3^ w v^−1^ in hexane for the neuronal identification study. For concentration-response analyses, dilutions ranged from 10^−8^ to 10^−2^ w v^−1^. Eight chemicals (phenyl acetic acid, acetic acid, 2,3-butadiol, 2,3-butadione, ethanol, 1,4-diaminobutane, geosmin, acetoin) were not sufficiently soluble and were dissolved in double-distilled water instead. Water and hexane were then used as control stimuli. Chemicals were prepared and administered as described in [39,40].

#### (iii) Cluster analysis and topographic mapping of sensilla

Hierarchical cluster analysis (Ward's method, squared Euclidean distance, version 21, IBM SPSS) was used as described [34,43] to identify OSN response classes. Raw responses to the chemical panel were corrected for solvent effects as discussed above and then *z*-transformed within sensilla to normalize the data. Corrected sensillum responses were divided by morphological class (large basiconic, small basiconic and trichoid), resulting in three separate cluster analyses. Discrete clusters were classified as different sensillum types as described previously [34]. OSN names were designated following standard *Drosophila* nomenclature (sensillum type and number followed by a letter designating OSN within that sensillum).

During recording, the morphological class of each sensillum was visually noted and its location sketched. After classification, we mapped and colour-coded the positions of sensilla on a template antenna. We then performed a MANOVA, using SPSS software, followed by the post-hoc Tukey's test to compare the locations of particular sensillum types. Positioning of sensillum types was also investigated in relation to host odour blends using a MANOVA.

#### (iv) Comparison of neuronal ensembles responding to various host blends

Venn diagrams were constructed to compare the overlap of response profiles of OSNs (as listed in full in the electronic supplementary material, figure S2 and table S2) to the volatile fruit blends for apple and downy hawthorn hosts attacked by *R. pomonella* races in the northeastern USA, as well as various other native hawthorn species infested by the fly in the southern USA outside the range of the apple fly (table 1). The mayhaw blend used for the Venn diagram was a combination of the three mayhaw varieties presented in table 1, as they are highly similar. We also removed butyl hexanoate from consideration in the downy hawthorn blend, as it is only present in trace amounts [13,15], and at concentrations found in the apple volatile blend butyl hexanoate is an antagonist to hawthorn flies [14].

## 3. Results

### (a) Scanning electron microscopy of antenna

**Figure 1.**
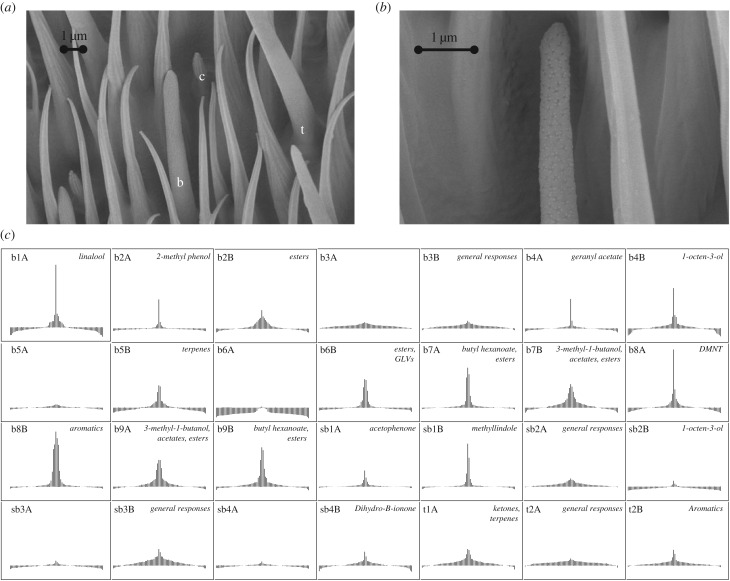
Classes of OSNs identified by SSR. (*a*) Scanning electron micrograph of the *R. pomonella* antenna. The three morphological types of olfactory sensilla recorded with SSR are shown: t, trichoid; b, basiconic; c, coeloconic. (*b*) Higher magnification micrograph of a basiconic sensilla showing the network of pores for odour molecule entry, visible at 10 000 times magnification. For (*a,b*), the scale is shown in micrometres. (*c*) Specificity curves summarizing the response profiles of *Rhagoletis* OSNs to the 76 odour panel, as constructed from the average response profiles in the electronic supplementary material, figure S2. Key ligands or ligand classes for each neuron are shown in italics. Neurons with no ligands listed are colocalized neurons showing no average response over 15 spikes s^−1^ within 1 s.e.m.

The antennal morphology of *Rhagoletis* was similar to that observed for *Drosophila* [19]. Mechanosensory hairs on the antennae observed in SEM were dense, with chemosensory sensilla generally shorter and more widely dispersed throughout the third segment of the antenna (electronic supplementary material, figure S1*a*). The three morphological classes of chemosensory sensilla were also apparent in *R. pomonella*: basiconic, trichoid and coeloconic (figure 1*a*). The rest of this study focuses on basiconic (figure 1*b*; electronic supplementary material, figure S1*b*) and trichoid sensilla, which in *Drosophila* are known to contain olfactory receptor cells [20].

### (b) Identification of olfactory sensory neuron classes based on single sensillum recordings

*Rhagoletis* exhibited highly specialized OSN classes tuned to specific host or non-host volatiles (figure 1*c*). Single sensillum electrophysiology yielded a total of 173 recordings from apple race flies to the complete panel of 76 odorants, allowing these OSNs to be categorized into classes based on their response profiles. In 11 of the 173 sensilla for which complete recordings were made (6%), none of the OSNs present responded to any odorant at more than 15 spikes s^−1^, and these sensilla were excluded from further analysis. Cluster analyses of the 162 responding sensilla resolved a total of 28 different OSN classes, 17 of which were housed in large basiconic (b) sensilla, eight in small basiconic (sb) sensilla and three in trichoid (t) sensilla (figure 1*c*; electronic supplementary material, figure S2 and table S2).

Recordings performed using a subset of 20 chemicals on hawthorn flies supported the implications of previous studies [34] that the host races do not differ in the OSNs they possess (figure 1*c*; electronic supplementary material, figure S2 and table S2). The 12 OSNs recorded in hawthorn flies were all correspondingly placed into one of the 28 different classes identified in the apple race, with responses to main ligands in the hawthorn race closely matching the apple race (electronic supplementary material, figure S3).

Most of the 28 different OSNs identified in apple flies showed high specificity for compounds within single chemical and/or ecological classes. Several *Rhagoletis* OSNs responded predominantly to only one odorant, such as b1A to linalool, b4B to 1-octen-3-ol and b8A to DMNT (figure 1*c*; electronic supplementary material, figure S2). With few exceptions, OSN responses were contained within a single chemical moiety, such as b7A and b9B, which both responded only to esters. Five OSNs (b3A, b5A, b6A, sb3A and sb4A) did not respond above 15 spikes s^−1^ to any chemical presented. However, these five non-responding OSNs could be distinguished by their colocalized OSN partner, which responded to at least one of the 76 tested compounds. OSNs could also be classified based on their selectivity for volatiles from different ecological sources (electronic supplementary material, table S1). A total of 29% (8/28) of the OSN classes responded exclusively to compounds from fruit sources. Another 29% (8/28) responded to compounds only from non-fruit sources (e.g. green leaves, microbes). Only 11% (3/28) of OSNs showed responses to both fruit and non-fruit sources. The remaining 32% of OSNs either did not respond to any tested compound (5/28) or gave weak responses to most volatiles tested (e.g. sb2A, sb3b; 4/28).

**Figure 2.**
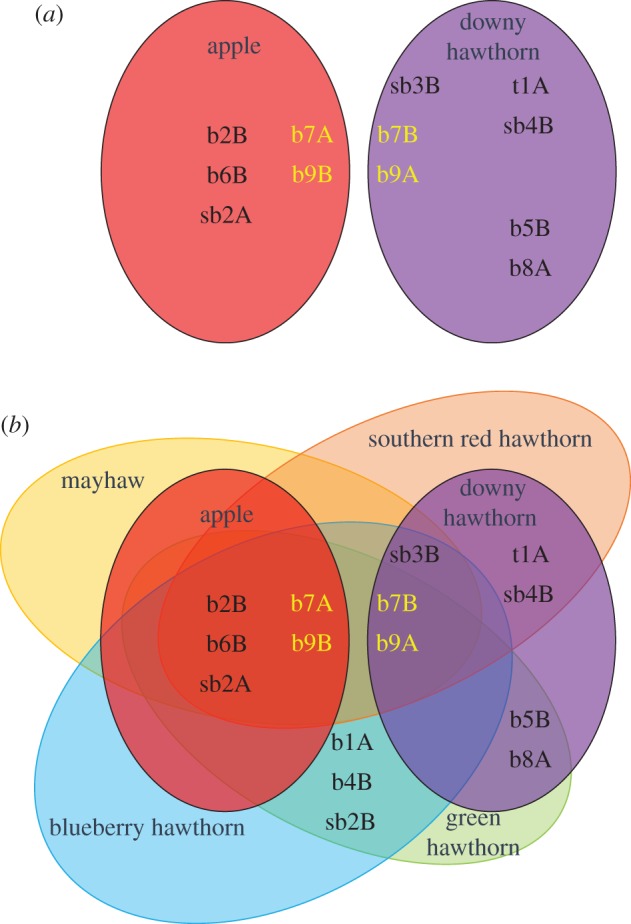
Projected overlap in OSN ensembles responding to host fruit blends. Diagrams were constructed based on the OSN response profiles in figure 1 and electronic supplementary material, figure S2 and table S2, and compared with the host fruit blends summarized in table 1. (*a*) Lack of OSN response overlap between the most recently diverged apple and downy hawthorn-infesting races of *R. pomonella*. (*b*) OSN response overlap when including southern United States hawthorn host races. Key neurons in sensilla b7 and b9, containing colocalized OSNs distinguishing apple and hawthorn blends, are highlighted in yellow.

There was no overlap in the OSNs responding to the behaviourally active volatiles in the apple versus downy hawthorn blends (figure 2*a*). The lack of overlap was primarily due to the absence of significant concentrations of esters in the downy hawthorn blend, which dominate the apple blend (table 1). The OSNs responding to esters found in the apple blend (b2B, b6B, b7A, b9B and sb2A) did not respond to any volatile in the downy hawthorn blend. Conversely, none of the OSNs that responded to volatiles found in the downy hawthorn blend (b5B, b7B, b8A, b9A, sb3B, sb4B and t1A) were sensitive to any volatile in the apple blend. Thus, the set of neurons tuned to elements of the apple versus downy hawthorn fruit blends differed in *R. pomonella*.

### (c) Ecological segregation of olfactory sensory neurons in sensilla

**Figure 3.**
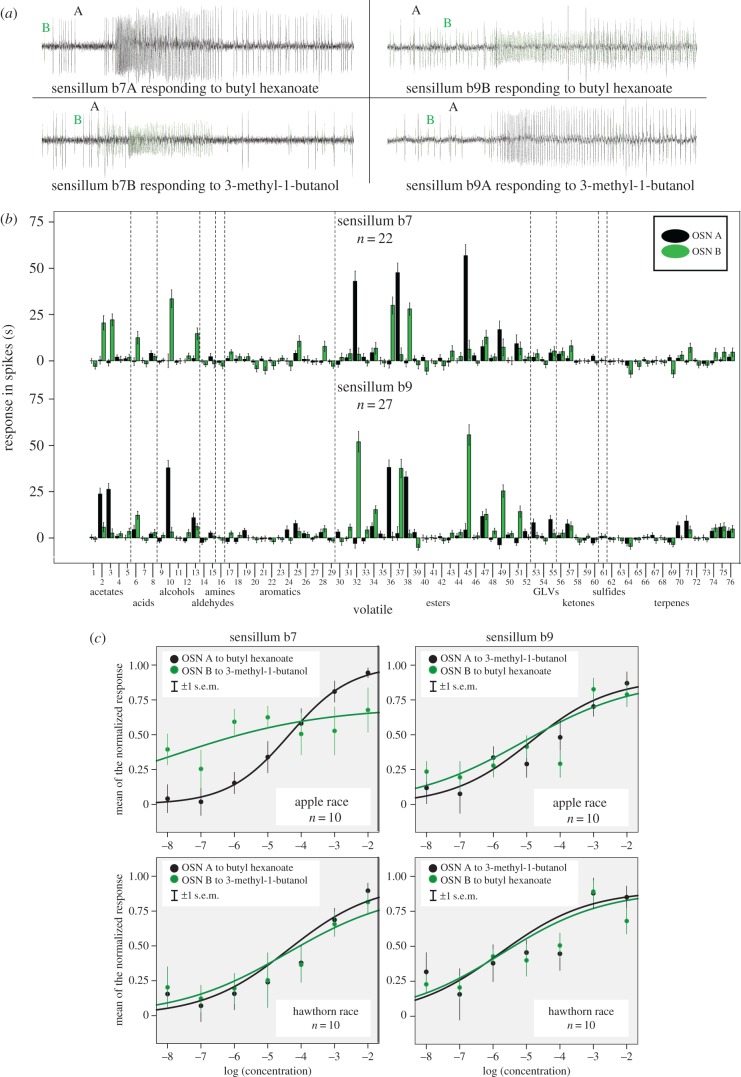
Response of OSNs in sensilla b7 and b9. (*a*) Raw spike recordings from b7 and b9 basiconic sensilla 1 s before and 2 s after presentation of the listed odorant. Individual OSNs are identified by spike amplitude size, with the large amplitude OSN labelled ‘A’ in black and the small labelled ‘B’ in green. (*b*) Complete response profiles for OSNs in sensilla b7 and b9 to the panel of 76 volatiles (designated by numbers) tested in the study. See the electronic supplementary material, table S1 for list of the compounds designated by the numbers. Average solvent corrected responses (±1 s.e.m.) in spikes s^−1^ are shown with the responses of the A neuron as black bars and the B neuron in green. (*c*) Concentration-response curves for OSNs in sensilla b7 and b9 in apple and hawthorn flies responding to butyl hexanoate or 3-methyl-1-butanol. Solvent-corrected, normalized, average responses (±1 s.e.m.) are shown, with the ‘A’ neuron in black and the ‘B’ in green, responding to their key ligand (either butyl hexanoate or 3-methyl-1-butanol). Responses were normalized prior to averaging by dividing each neuron's sequence of responses by the highest response observed for that neuron with lines fitted by logistic curve estimation. (Online version in colour.)

In *Rhagoletis*, the 28 identified OSN classes were organized into 15 different stereotyped combinations in sensilla. Fruit volatile-responding OSNs were generally segregated from one another in different sensilla types and colocalized with a non-fruit responder. The only exceptions were sensilla b7 and b9. For these two sensilla, the large-spiking ‘A’ neuron in b7 and small-spiking ‘B’ neuron in b9 responded to three esters in the apple blend, including the major behaviourally active ester butyl hexanoate, while their accompanying OSNs b7B and b9A, respectively, responded to the key downy hawthorn volatile 3-methyl-1-butanol, in addition to isoamyl acetate (figure 3). As these four colocalized OSNs responded to the key behavioural agonist and antagonist for each race, we tested the sensitivity of these OSNs to different concentrations of butyl hexanoate and 3-methyl-1-butanol for possible differences between the host races.

### (d) Concentration-response sensitivity of sensillum types b7 and b9

The overall sensitivities of OSNs in sensilla b7 and b9 to the key volatiles butyl hexanoate and 3-methyl-1-butanol did not differ between apple and hawthorn flies when tested across a range of concentrations from 10^−8^ to 10^−2^ w v^−1^ (*t*-test, *p* > 0.05; figure 3*c*). The only exception was the response of the b7B neuron to 3-methyl-1-butanol at a concentration 10^−6^, where the apple race response was significantly higher than that of hawthorn flies (figure 3*c*). This could be due to the inherent difficulty in separating the small B neuron spikes in these sensilla. However, there was no difference between apple and hawthorn neurons at higher concentrations (10^−5^ through 10^−2^). Consequently, apple and hawthorn flies share the same colocalized OSNs in sensilla b7 and b9, and these paired neurons exhibit similar sensitivities to the major attractant and antagonist fruit volatiles between the host races.

### (e) Spatial distribution of host volatile-responding olfactory sensory neurons on antenna

Topographic maps showed no significant difference in the spatial distribution of sensilla responding to components of the apple and downy hawthorn fruit blends on the antennae of flies (MANOVA *p* > 0.05; electronic supplementary material, figure S4*b,c*). There was, however, a significant difference in the distribution of sensilla sb1 and b4 along the *y*-axis of the antenna (*p* < 0.05, as determined by MANOVA with post-hoc Tukey's tests) due to these two sensilla being located in the distal half of the antenna (electronic supplementary material, figure S4*a*). However, these OSNs did not respond to apple or downy hawthorn volatiles (figure 1*c*; electronic supplementary material, figure S2 and table S2), and therefore their positioning does not affect apple and hawthorn fly host-seeking behaviour.

## 4. Discussion

This study characterized patterns of OSN specificity, sensitivity and organization in *R. pomonella* to identify possible neuronal pathways responsible for fruit odour discrimination between apple and downy hawthorn-infesting host races of the fly. Initially, our results suggest that no major difference exists in the classes, sensitivities or spatial distribution of antennal OSNs. However, several features of the *R. pomonella* peripheral olfactory system indicate that alterations in specific neuronal pathways (channels) for apple and downy hawthorn volatile detection could potentially result in dramatic shifts in host fruit odour discrimination. First, only a limited number of OSNs respond to the behaviourally active components of the apple and hawthorn fruit blends. Second, there is no overlap in the OSNs responding to the active components of the apple versus downy hawthorn blends, with specific sets of OSNs responding to the behaviourally relevant compounds in either apple or downy hawthorn fruit. This indicates that only a few neurons are involved in host-seeking behaviour. Third, the two most behaviourally important volatiles in the apple and downy hawthorn blends (butyl hexanoate and 3-methyl-1-butanol, respectively) are primarily recognized by just four OSNs colocalized in attractant and antagonist-responding pairs in two sensilla (b7 and b9). Thus, a change in the butyl hexanoate and 3-methyl-1-butanol pathways in these sensilla could rapidly shift fruit odour discrimination of apple and hawthorn flies from attraction to antagonism and vice versa.

There are several different mechanisms through which such a change in behaviour could occur (see the electronic supplementary material, figure S5). For example, a swap in olfactory receptor expression between these paired neurons in sensilla, as proposed for sex pheromone OSNs in the European corn borer [26,28], could flip the processing of attractant and antagonistic sensory signals. In a related manner, a switch in the projections of butyl hexanoate and 3-methyl-1-butanol OSNs to glomeruli in the antennal lobe would have similar consequences. Alternatively, alterations in central processing of olfactory cues through changes in synaptic transmission or connectivity of interneurons linking the OSN-corresponding glomeruli in the antennal lobe [44] or through levels of neuromodulators that shift, or react to shifts, in response thresholds [45] could also have similar consequences for behaviour. Further work on these neurons and their central targets is necessary to determine which of the above mechanisms or their combination is correct, or if the change in behaviour occurred by a different route altogether [21,46].

The discovery of butyl hexanoate and 3-methyl-1-butanol OSNs colocalized together in the sensilla of *R. pomonella* is also intriguing. In this regard, similar colocalization of behaviourally relevant OSNs has been reported in the sex pheromone systems of several moth species (e.g. *Ostrinia nubilalis* [25–29], *Helicoverpa zea* [47,48] and *Spodoptera littoralis* [49]), as well as for sex pheromone and host volatile OSNs in a bark beetle [50], suggesting possible functional significance. Colocalization of neurons together in a single sensillum is believed to enhance coincidence detection of individual compounds, improving resolution of odour blends, and distinguishing them from both background odours and potential antagonists [50–52]. It is therefore possible that colocalization of neurons responding to attractant and antagonist host volatiles may have contributed to the behavioural divergence of apple and hawthorn flies by providing a physiological mechanism for enhancing the detection and discrimination of the fruit blends for each race.

It also remains to be determined why there are dedicated neuronal pathways for esters in downy hawthorn flies, when these compounds are essentially absent from the downy hawthorn fruit blend and downy hawthorn flies do not depend on them for attraction to their host (table 1). Part of the answer may lie in the observation that all five of the esters present in the apple blend are found in varying combinations along with 3-methyl-1-butanol in different native hawthorn species attacked by *R. pomonella* in the southern USA (table 1). In particular, the fruit blends for mayhaw, blueberry hawthorn, green hawthorn and southern red hawthorn all contain a relatively high proportion of butyl hexanoate, the primary attractant for apple flies. Thus, there is an evolutionary legacy for why OSNs for butyl hexanoate and 3-methyl-1-butanol could be important as pathways for information prompting specific behaviours; both serve in the recognition of southern hawthorn fruit. This does not explain how butyl hexanoate evolved to act as an antagonist for northern downy hawthorn flies, when it is not for southern hawthorn flies [32,53]. It may be that in the northeastern USA the compound is associated with a less suitable host fruit for fly survivorship, such as various native crabapple species that are absent from the south [33], and thus butyl hexanoate was selected to be a behavioural antagonist.

## 5. Conclusion

We found specific, dedicated neurons that act as specialized pathways for olfactory ecological information and could facilitate host plant shifts and incipient speciation in *Rhagoletis* flies. Relatively simple shifts in neuronal pathways carrying information of behavioural relevance may represent a general phenomenon contributing to rapid shifts in habitat and mate choice, potentially catalysing speciation. Behaviour is ‘the pacemaker of evolution’ [1], but it may be the presence of strongly ecologically tuned sensory pathways and the way in which they are organized that provide the means for behavioural change.

## Supplementary Material

Sensory specificity and speciation: A potential neuronal pathway for host fruit odor discrimination in Rhagoletis pomonella - Tait et al. Electronic Supplementary Materials

## Data accessibility

The datasets supporting this article have been uploaded as part of the electronic supplementary material.
