# Supplementary material for: Sensory specificity and speciation: a potential neuronal pathway for host fruit odour discrimination in Rhagoletis pomonella
Source: Proc Biol Sci. 2016 Dec 28;283(1845):20162101. doi: 10.1098/rspb.2016.2101 (PMC5204164; doi:10.1098/rspb.2016.2101)
Supplement: Sensory specificity and speciation: A potential neuronal pathway for host fruit odor discrimination in Rhagoletis pomonella - Tait et al. Electronic Supplementary Materials [file rspb20162101supp1.pdf]

Sensory specificity and speciation: A potential neuronal pathway for host fruit odor discrimination in *Rhagoletis pomonella*

Electronic Supplementary Materials

Table s1. Summary of the 76 chemicals used in SSR panel. Compounds are listed by chemical moiety, common name, ID number, CAS number, manufacturer, purity, major ecological sources as obtained from https://pubchem.ncbi.nlm.nih.gov/, and inclusion in other species panels as mentioned in the text (*Rhagoletis*: Zhang et al. 1999, Nojima et al. 2003, Linn et al. 2003, Cha et al. 2011a, 2011b; *Bactrocera*: Biasazin et al. 2014; *Drosophila*: Hallem and Carlson 2006).

Moiety	Compound	ID # in this study	CAS #	Company	Purity	Ecological source	Tested with species
Acetate	Methyl acetate	1	79-20-9	FLUKA	99.8%	Fruits	*Drosophila*
Acetate	Pentyl acetate	2	628-63-7	Aldrich	99%	Fruits	*Rhagoletis, Drosophila*
Acetate	Isoamyl acetate	3	123-92-2	Aldrich	99%	Fruits	*Rhagoletis, Bactrocera, Drosophila*
Acetate	Hexyl acetate	4	142-92-7	Aldrich	99%	Plant volatile	*Rhagoletis, Bactrocera, Drosophila*
Acetate	Ethyl acetate	5	141-78-6	Aldrich	99.8%	microbes, plant volatile	*Rhagoletis, Drosophila*
Acetate	Butyl acetate	6	123-86-4	FLUKA	99%	Fruits	*Rhagoletis, Bactrocera, Drosophila*
Acid	Acetic acid	7	64-19-7	Aldrich	99.9%	Microbes	*Drosophila*
Acid	Hexanoic acid	8	142-62-1	Aldrich	99.5%	Microbes	*Drosophila*
Acid	Phenyl acetic acid	9	103-82-2	Aldrich	99%	Plant oils	
Alcohol	3-methyl-1-butanol	10	123-51-3	Sigma	99%	Fruits, microbes, plant volatile	*Rhagoletis, Bactrocera, Drosophila*
Alcohol	1-octen-3-ol	11	3391-86-4	SAFC	98%	Fruits, mushrooms	*Rhagoletis, Drosophila*
Alcohol	2,3-butadiol	12	513-85-9	FLUKA	99%	Microbes	*Drosophila*
Alcohol	1-hexanol	13	111-27-3	FLUKA	99%	Plant oils	*Drosophila*
Alcohol	Ethanol	14	64-17-5	Sigma	99.5%	Microbes	*Drosophila*
Aldehyde	Nonanal	15	124-19-6	FLUKA	95%	Fruits, plant volatile	*Rhagoletis*
Aldehyde	Decanal	16	112-31-2	FLUKA	95%	Fruits	
Amine	1,4-diaminobutane	17	110-60-1	Aldrich	99%	Microbes	*Drosophila*

Aromatic	Geosmin	18	16423-19-1	Sigma	97%	Microbes	*Drosophila*
Aromatic	p-cymene	19	99-87-6	Aldrich	99%	Trees	*Drosophila*
Aromatic	Phenyl ethyl alcohol	20	60-12-8	FLUKA	99%	Plant oils	*Drosophila*
Aromatic	Phenyl acetaldehyde	21	122-78-1	Aldrich	90%	Flowers, plant volatile	*Drosophila*
Aromatic	Methylindole	22	603-76-9	Aldrich	97%	Flowers	
Aromatic	Methyl salicylate	23	119-36-8	Sigma	99%	Trees	*Drosophila*
Aromatic	2-methyl phenol	24	95-48-7	FLUKA	99.5%	Microbes, plant oils	*Drosophila*
Aromatic	Benzyl alcohol	25	100-51-6	Aldrich	99.8%	Flowers, trees	*Drosophila*
Aromatic	Ethyl benzoate	26	93-89-0	Aldrich	99%	Plant volatile	*Drosophila*
Aromatic	Benzaldehyde	27	100-52-7	Sigma	99%	Plant volatile	*Drosophila*
Aromatic	Acetophenone	28	98-86-2	FLUKA	99%	Plant oils, trees, mosses	*Drosophila*
Aromatic	Cyclohexane	29	108-94-1	Sigma	99.8%	Plant volatile	
Ester	2-pentyl butyrate	30	60415-61-4	Aldrich	99%	Fruits	*Rhagoletis, Bactrocera*
Ester	Methyl octanoate	31	111-11-5	FLUKA	99%	Fruits	*Drosophila*
Ester	Propyl hexanoate	32	626-77-7	Aldrich	98%	Fruits	*Rhagoletis*
Ester	Methyl hexanoate	33	106-70-7	Aldrich	99.8%	Fruits	*Bactrocera, Drosophila*
Ester	Butyl propionate	34	590-01-2	Alfa Aesar	99%	Fruits	*Drosophila*
Ester	Methyl benzoate	35	93-58-3	Aldrich	99%	Microbes, plant oils	*Drosophila*
Ester	Isoamyl butyrate	36	106-27-4	Aldrich	98%	Fruits	*Rhagoletis, Bactrocera*
Ester	Pentyl hexanoate	37	540-07-8	Aldrich	98%	Fruits	*Rhagoletis*
Ester	Isoamyl hexanoate	38	2198-61-0	Merck	98%	Microbes, fruits	
Ester	Hexyl isobutyrate	39	2349-07-7	SAFC	97%	Plant volatile	*Drosophila*
Ester	Ethyl tiglate	40	5837-78-5	Aldrich	98%	Flowers	*Bactrocera*
Ester	Ethyl crotonate	41	623-70-1	Aldrich	99%	Microbes, fruits	*Bactrocera, Drosophila*
Ester	Hexyl butyrate	42	2639-63-6	Aldrich	98%	Fruits	*Rhagoletis, Drosophila*

Ester	Ethyl octanoate	43	106-32-1	Aldrich	98%	Fruits	*Drosophila*
Ester	Ethyl butyrate	44	105-54-4	Sigma	99%	Fruits	*Bactrocera, Drosophila*
Ester	Butyl hexanoate	45	626-82-4	TCI	98%	Fruits	*Rhagoletis*
Ester	Ethyl lactate	46	97-64-3	Aldrich	98%	Plant and animal metabolite	*Drosophila*
Ester	Butyl butanoate	47	109-21-7	FLUKA	99%	Fruits	*Rhagoletis, Bactrocera*
Ester	Ethyl isovalerate	48	108-64-5	FLUKA	99%	Fruits	*Bactrocera*
Ester	Ethyl hexanoate	49	123-66-0	Aldrich	99%	Fruits	*Bactrocera, Drosophila*
Ester	Ethyl-3-hydroxybutyrate	50	5405-41-4	FLUKA	97%	Fruits	*Drosophila*
Ester	Ethyl heptanoate	51	106-30-9	FLUKA	99%	Fruits	*Rhagoletis, Drosophila*
Ester	Hexyl propionate	52	2445-76-3	SAFC	97%	Fruits	*Rhagoletis, Drosophila*
GLV	z-3 hexenyl acetate	53	3681-71-8	Aldrich	98%	Fruits	*Rhagoletis, Bactrocera*
GLV	E2 hexenyl acetate	54	2497-18-9	Aldrich	98%	Plant volatile	*Drosophila*
GLV	E2 hexenol	55	928-95-0	FLUKA	95%	Plant volatile	*Drosophila*
Ketone	6-methyl-5-hepten-2-one	56	110-93-0	Aldrich	99%	Fruits	*Drosophila*
Ketone	2-heptanone	57	110-43-0	Aldrich	98%	Plant oils	*Bactrocera, Drosophila*
Ketone	Dihydro-beta-ionone	58	17283-81-7	Aldrich	90%	Fruits	*Rhagoletis*
Ketone	Acetoin	59	513-86-0	SAFC	96%	Microbes	*Drosophila*
Ketone	2,3-butadione	60	431-03-8	FLUKA	99.4%	Microbes, plant oils	*Drosophila*
Sulfide	Dimethyl trisulfide	61	3658-80-8	Aldrich	98%	Fruits, Microbes	*Rhagoletis, Drosophila*
Terpene	Ocimene	62	13877-91-3	Aldrich	90%	Flowers	*Bactrocera*
Terpene	Terpinolene	63	586-62-9	SAFC	90%	Trees	*Drosophila*
Terpene	Nerol	64	106-25-2	Aldrich	97%	Plant volatile	*Drosophila*
Terpene	Farnesol	65	4602-84-0	Aldrich	95%	Flowers	
Terpene	Sabinene	66	3387-41-5	ROTH	97%	Plant volatile	

Terpene	Dmnt	67	51911-82-1	From Bartram, MPI CE	95%	Fruits, plant volatile	*Rhagoletis*
Terpene	Racemic limonene	68	(+)5989-27-5 (-)5989-54-8	FLUKA Aldrich	98% 96%	Fruits, plant volatile	*Rhagoletis, Bactrocera, Drosophila*
Terpene	Citral	69	5392-40-5	Aldrich	95%	Fruits	*Drosophila*
Terpene	Beta-myrcene	70	123-35-3	FLUKA	95%	Fruits	*Bactrocera, Drosophila*
Terpene	Alpha-pinene	71	80-56-8	Aldrich	98%	Plant volatile	*Bactrocera, Drosophila*
Terpene	3-carene	72	13466-78-9	Aldrich	90%	Plant oils	*Bactrocera, Drosophila*
Terpene	Geranyl acetate	73	105-87-3	Sigma	98%	Plant oils	*Drosophila*
Terpene	Beta-pinene	74	127-91-3	ROTH	97%	Trees	*Bactrocera, Drosophila*
Terpene	Beta-caryophyllene	75	87-44-5	FLUKA	98%	Fruits, plant oils	*Drosophila*
Terpene	Linalool	76	78-70-6	Aldrich	97%	Fruits, plant volatile	*Rhagoletis, Bactrocera, Drosophila*

Table s2. Summary of strongest *R. pomonella* OSN responses. This table summarizes information displayed by the graphs of Figure s2, showing first all the sensillar morphologies seen, then the sensilla types, then the “A” or “B” neurons they contain, then their responses to all of the ligands responded to in this study. Intensities of response are shown by numbers of (*) in order of increasing strength: 1 = a response of 15-30 spikes/sec, 2 = a response of 30-60 spikes/sec, 3 = a response greater than 60 spikes/sec. Also shown are the ecological groups of the ligands: apple or downy hawthorn fruit blends, general fruit volatiles, general plant volatiles, or microbial volatiles.

	Morphology	Basiconic	Basiconic		Basiconic		Basiconic		Basiconic		Basiconic		Basiconic		Basiconic		Basiconic	
	Sensillum	b1	b2		b3		b4		b5		b6		b7		b8		b9	
	OSN	A	A	B	A	B	A	B	A	B	A	B	A	B	A	B	A	B
Apple or downy hawthorn host fruit volatiles	Butyl butanoate			*														
	Propyl hexanoate			*								*	**					***
	Butyl hexanoate			*									***					***
	Hexyl butyrate			**														
	Pentyl hexanoate			*									**					**
	3-methyl-1-butanol													**			**	
	DMNT									**					***			
	Isoamyl acetate													*			*	
	Di-hydro-beta-ionone																	
Fruit volatiles	Isoamyl hexanoate													**			**	
	Pentyl acetate													*			*	
	Isoamyl butyrate													**			**	

Fruit volatiles continued	1-octen-3-ol	*						***										
	Hexyl propionate			*														
	Butyl propionate																	*
	2-pentyl butyrate			*														
	Ethyl heptanoate			*								**						*
	Ethyl hexanoate			*								**	*					*
	Ethyl octanoate			*														
	Citral														**			
	Beta-myrcene	*																
	Linalool	***								**								
	Limonene	*																
	Nonanal																	
Plant volatiles	Terpinolene	*																
	Ocimene	*								**								
	3-carene						*											
	Geranyl acetate						**								*			
	Nerol																	
	E2 hexenyl acetate											**						
	E2 hexenol							*										
	1-hexanol							*						*				
	Ethyl-3-hydroxy butyrate							*										
	Acetophenone															***		
	Benzaldehyde															***		
	Benzyl alcohol															***		
	Methylindole															*		
	Phenylacetaldehyde															*		
	Phenyl ethyl alcohol															***		
	Hexyl acetate																	
	Methyl salicylate																	
	Ethyl benzoate																	
Microbe volatiles	2-methyl-phenol		**			*										***		
	Hexanoic acid																	

	Morphology	Small basiconic		Small basiconic		Small basiconic		Small basiconic		Trichoid	Trichoid	
	**Sensillum**	**sb1**		**sb2**		**sb3**		**sb4**		**t1**	**t2**	
	**OSN**	**A**	**B**	**A**	**B**	**A**	**B**	**A**	**B**	**A**	**A**	**B**
Apple or downy hawthorn host fruit volatiles	Butyl butanoate											
	Propyl hexanoate											
	Butyl hexanoate											
	Hexyl butyrate			*								
	Pentyl hexanoate											
	3-methyl-1-butanol											
	DMNT											
	Isoamyl acetate											
	Di-hydro-beta-ionone						*		*	*		
Fruit volatiles	Isoamyl hexanoate											
	Pentyl acetate						*					

Fruit volatiles continued	Isoamyl butyrate											
	1-octen-3-ol				*							
	Hexyl propionate											
	Butyl propionate											
	2-pentyl butyrate											
	Ethyl heptanoate											
	Ethyl hexanoate											
	Ethyl octanoate											
	Citral						*		*	*		
	Beta-myrcene											
	Linalool											
	Limonene											
	Nonanal										*	
Plant volatiles	Terpinolene											
	Ocimene											
	3-carene											
	Geranyl acetate									*		
	Nerol									*		
	E2 hexenyl acetate											
	E2 hexenol						*					
	1-hexanol											
	Ethyl-3-hydroxy butyrate											
	Acetophenone	*										
	Benzaldehyde		*									
	Benzyl alcohol											
	Methylindole		***									
	Phenylacetaldehyde											
	Phenyl ethyl alcohol											
	Hexyl acetate											*
	Methyl salicylate											*
	Ethyl benzoate											*
Microbe volatiles	2-methyl-phenol		**		*							
	Hexanoic acid			*								

S. Figure Legends

Figure s1. Scanning electron micrographs of the *R. pomonella* antenna. Scales are shown in µm. (a) The three segments of the fly antenna, with the arista at top right and the eye at top, for orientation. (b) Higher magnification micrograph of a basiconic sensilla showing the network of pores for odour molecule entry, visible at 50,000 times magnification, with the approximate pore diameter in shown.

Figure s2. Profiles of *R. pomonella* antennal OSNs. Graphs show responses of colocalized neurons within basiconic "b" (b1-b9), small basiconic "sb" (sb1-sb4) and trichoid "t" sensilla (t1-t2). The X-axis lists the ID numbers of the 76 volatiles used as stimuli (Table s1); the Y-axis shows average response levels in spikes/second (error bars +/- 1 SEM) corrected for solvent effects. The chemical moiety of the 76 volatiles is indicated below the x-axis, and delineated by dotted lines through all graphs. The responses of the larger spiking “A” neuron are shown by black bars and the smaller

spiking “B” neuron in green. N represents the number of sensilla of each type recorded and averaged to produce the graphs.

Figure s3. Response profiles of OSNs in apple and hawthorn race flies. Graphs show responses of colocalized neurons within b1, b6, b7, b9, and sb1 sensilla between the apple and downy hawthorn host races. The X-axis shows the ID numbers of the reduced set of volatiles used as stimuli; the Y-axis depicts the average normalized responses corrected for solvent effects, and formed by dividing all raw spike/second responses by the average response of the largest magnitude. Again, the responses of the larger spiking “A” neuron are shown by black bars while the smaller spiking “B” neuron responses are shown in green. N represents the number of sensilla of each type recorded.

Figure s4. Topographic map showing spatial patterning of sensillum types. (a) The topographical position of all 15 sensilla types on the R. pomonella antenna, labelled by colour and shape as listed in the key. (b) Sensilla that responded to apple blend components highlighted in red. (c) Sensilla that responded to the downy hawthorn components highlighted in blue. No spatial patterns were found to be statistically significant (SPSS MANOVA, $p > 0.05$).

Figure s5. Proposed neuronal mechanisms underlying host shifts and speciation in the *Rhagoletis pomonella* host complex. Simplified for clarity, only one of the two sensilla is shown, containing two neurons, A the larger OSN and B the smaller OSN, with their odour receptor proteins shown in colours corresponding to what volatile each specifically fires in response to (red for butyl hexanoate and blue for 3-methyl-1-butanol). The information then travels through a “cloud” representing the brain and ultimately gives rise to attractive or antagonistic behaviours. A reversal in the coding of these compounds by the nervous system, either through switching (a) olfactory receptor protein expression, (b) sensory neuron targeting or (c) central processing within the brain via neuromodulation shifts or wiring of interneurons (mechanisms not shown) would switch attractants to antagonists. This would subsequently reverse behavioural response to the fruit odours coded by these sets of OSNs, allowing the ancestral hawthorn race to give rise to the apple race as indicated by the thin black directional arrows in panels a-c.

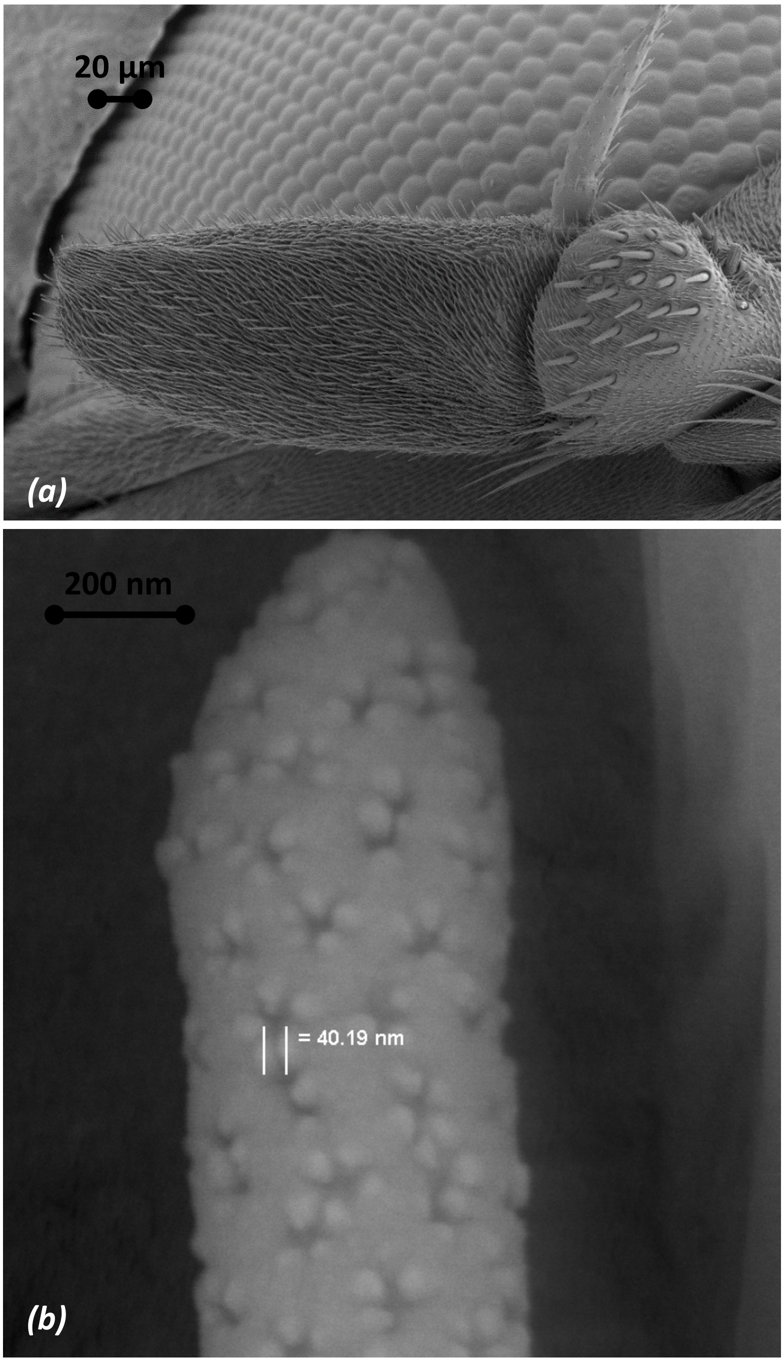

Figure s1.

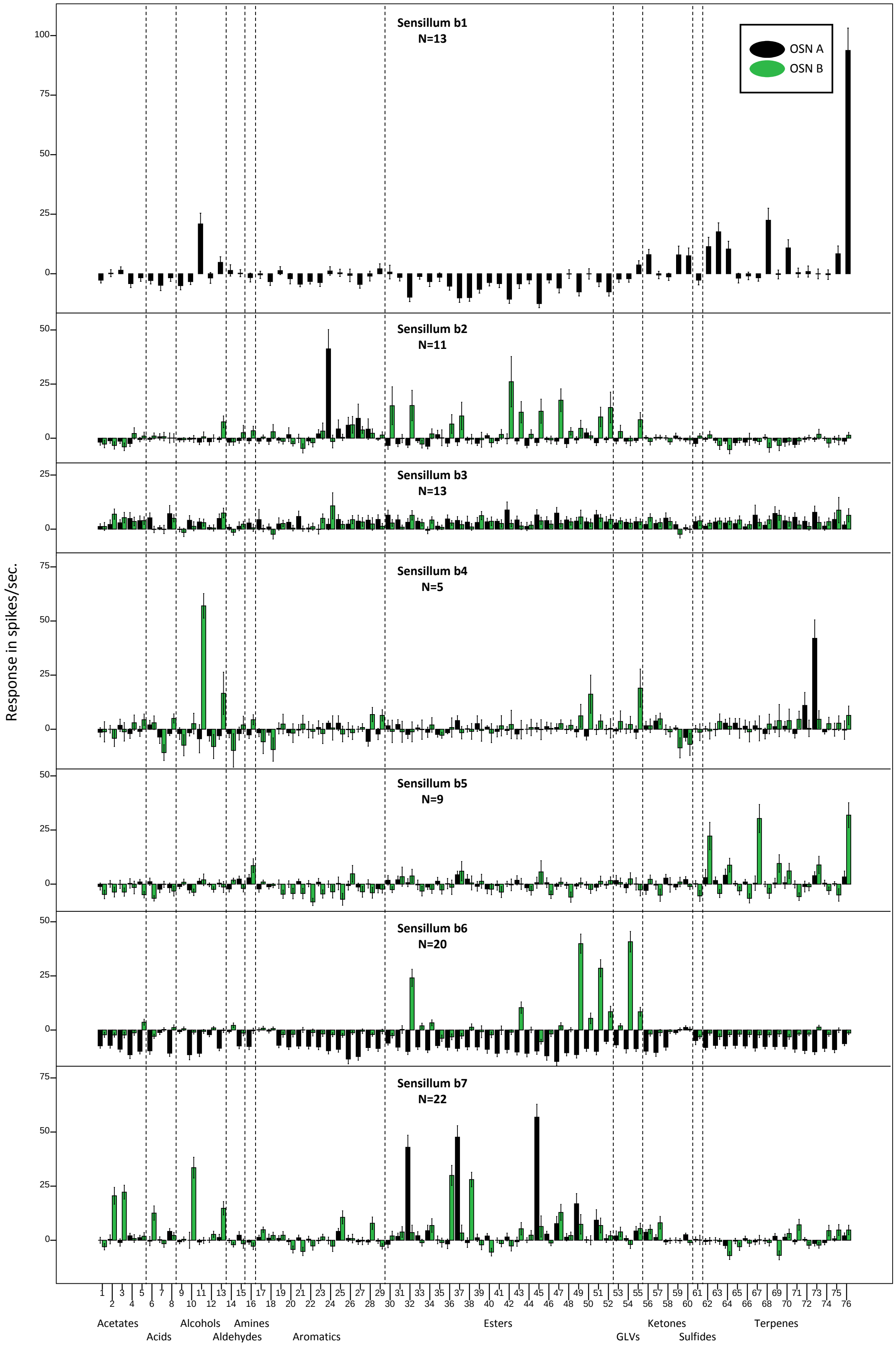

Sensillum b1
N=13
OSN A
OSN B
Sensillum b2
N=11
Sensillum b3
N=13
Sensillum b4
N=5
Sensillum b5
N=9
Sensillum b6
N=20
Sensillum b7
N=22
Response in spikes/sec.
Acetates
Acids
Alcohols
Aldehydes
Amines
Aromatics
Esters
GLVs
Ketones
Sulfides
Terpenes

Figure s2.

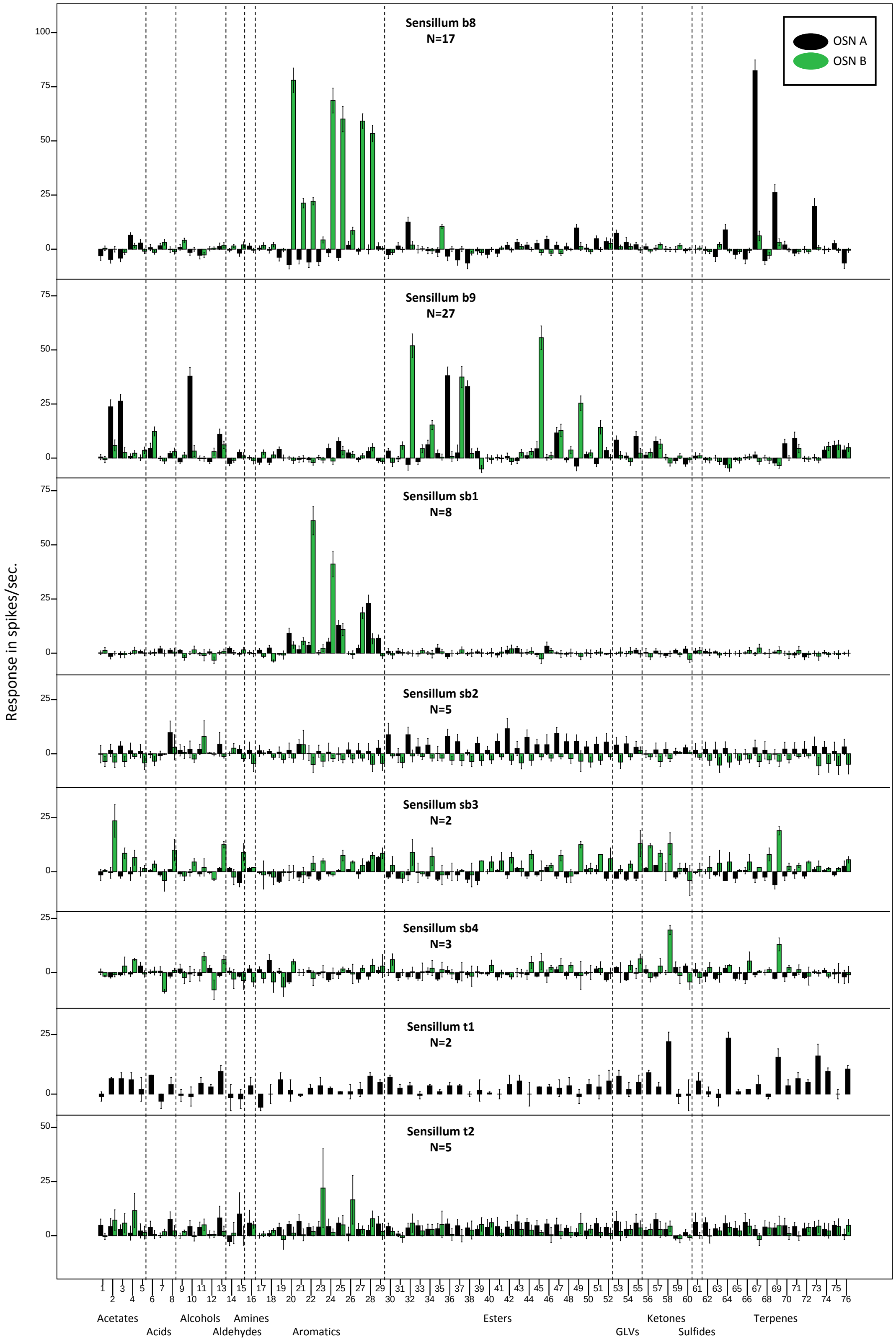

Figure s2 continued.

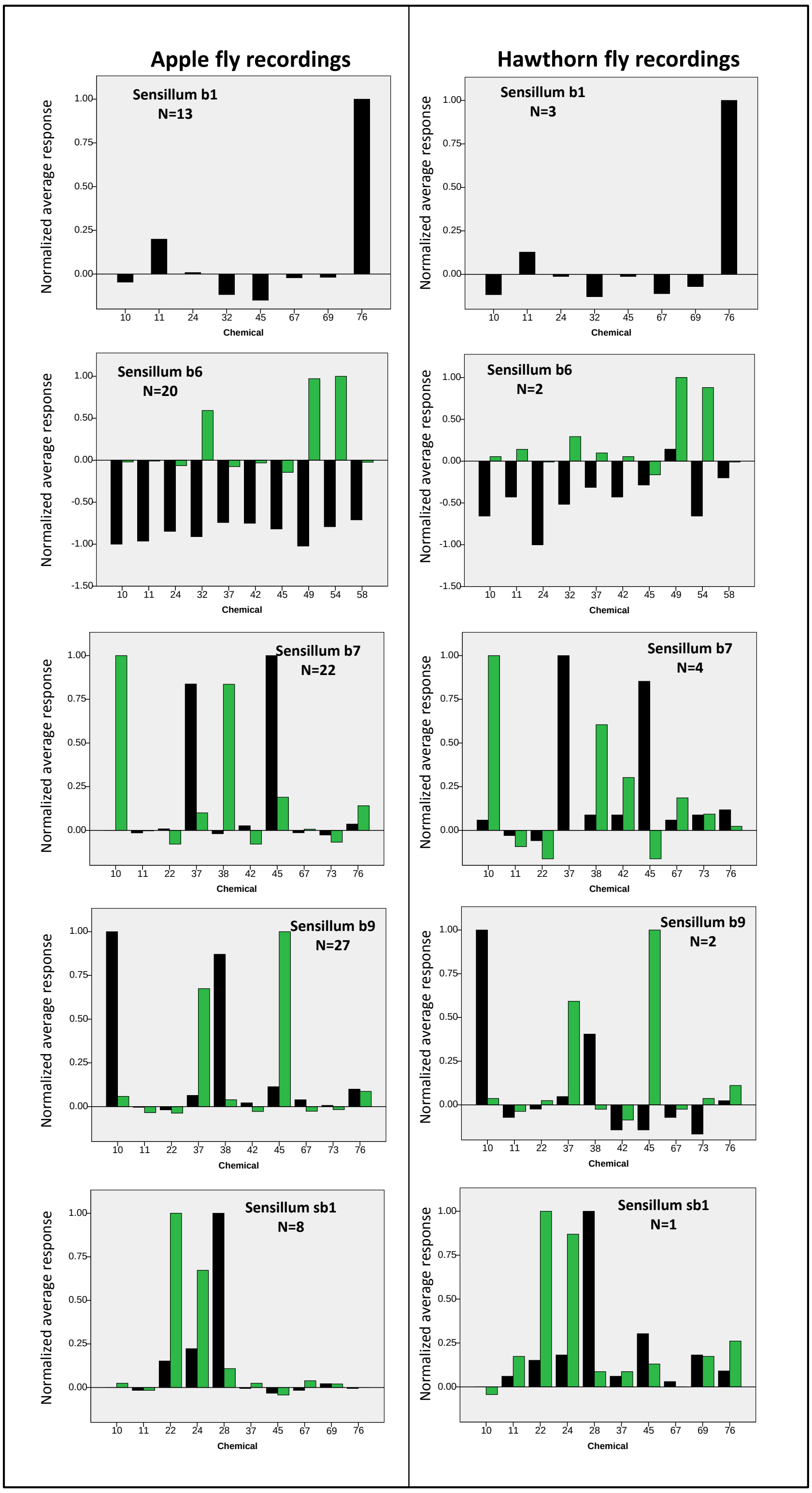
Apple fly recordings
Hawthorn fly recordings
Sensillum b1
N=13
Sensillum b1
N=3
Sensillum b6
N=20
Sensillum b6
N=2
Sensillum b7
N=22
Sensillum b7
N=4
Sensillum b9
N=27
Sensillum b9
N=2
Sensillum sb1
N=8
Sensillum sb1
N=1
Normalized average response
Chemical

Figure s3.

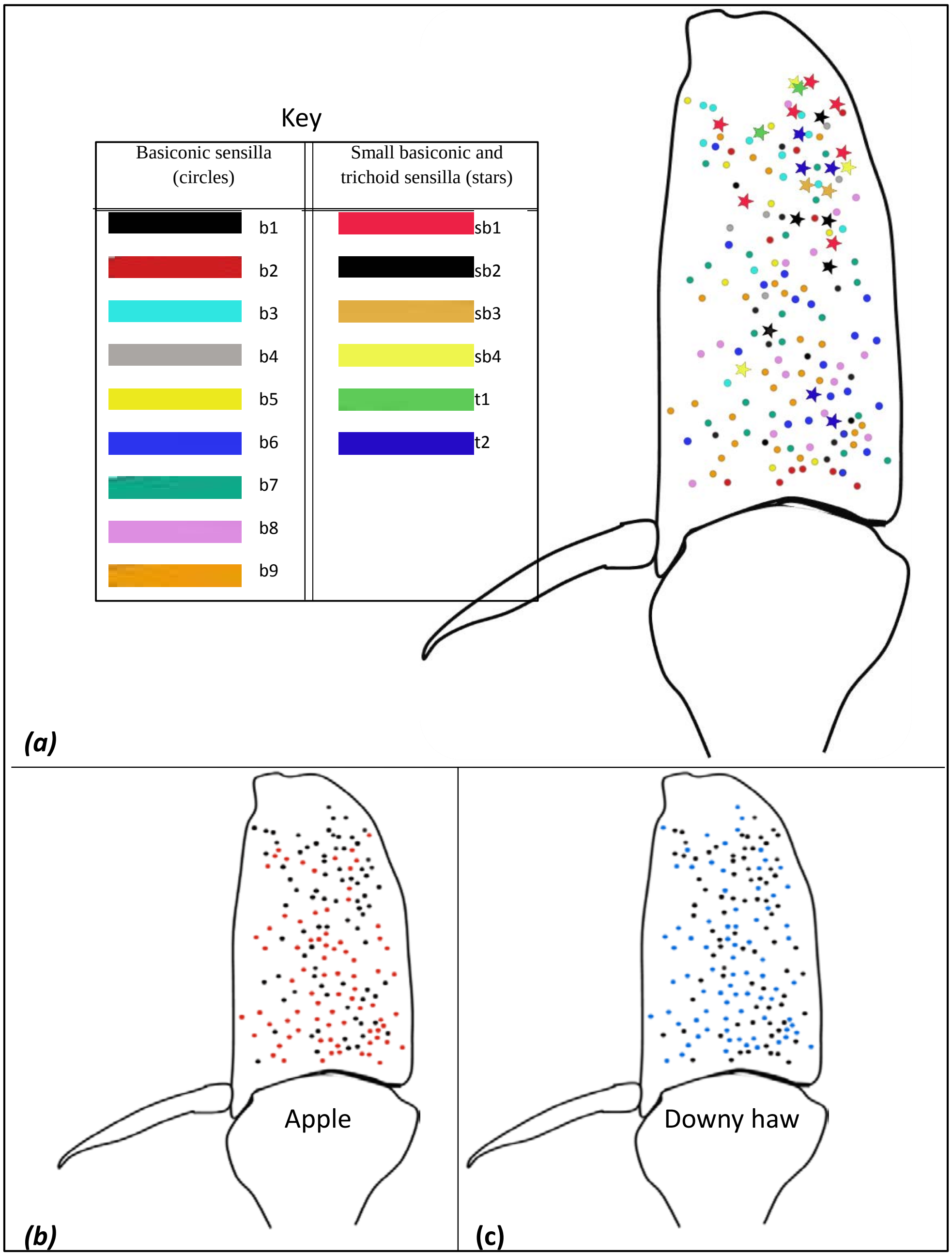
Key
Basiconic sensilla (circles)
Small basiconic and trichoid sensilla (stars)
b1
b2
b3
b4
b5
b6
b7
b8
b9
sb1
sb2
sb3
sb4
t1
t2
(a)
Apple
Downy haw
(b)
(c)

Figure s4.

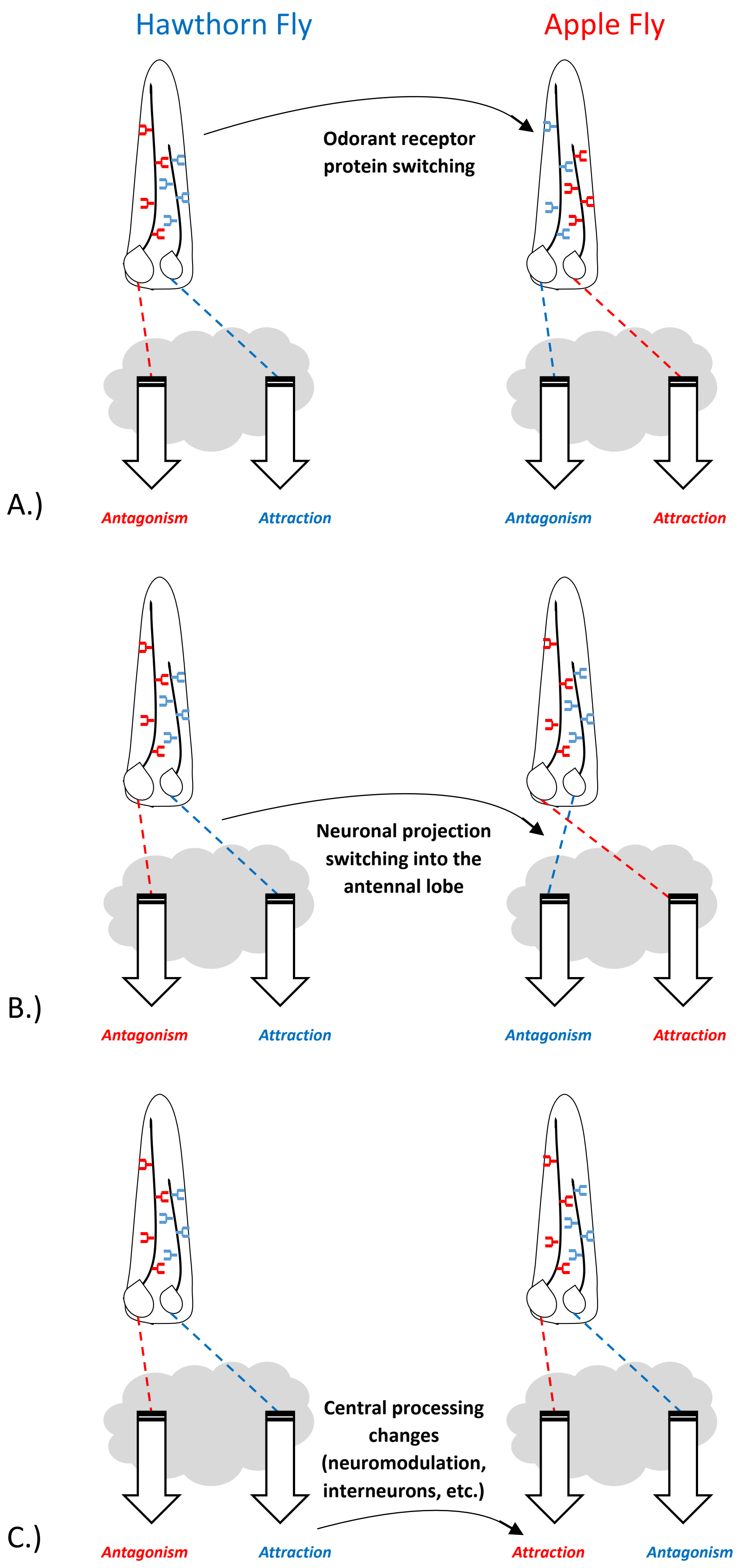
Hawthorn Fly
Apple Fly
Odorant receptor protein switching
A.)
Antagonism
Attraction
Antagonism
Attraction
Neuronal projection switching into the antennal lobe
B.)
Antagonism
Attraction
Antagonism
Attraction
Central processing changes (neuromodulation, interneurons, etc.)
C.)
Antagonism
Attraction
Attraction
Antagonism

Figure s5.
